# Saccadic trajectories deviate toward or away from optimally informative visual features

**DOI:** 10.1016/j.isci.2023.107282

**Published:** 2023-07-04

**Authors:** Serena Castellotti, Martin Szinte, Maria Michela Del Viva, Anna Montagnini

**Affiliations:** 1 University of Florence, Department of Neurofarba, Florence, Italy; 2 Institut de Neurosciences de la Timone, CNRS and Aix-Marseille Université, Marseille, France

**Keywords:** Sensory neuroscience, Cognitive neuroscience

## Abstract

The saccades’ path is influenced by visual distractors, making their trajectory curve *away* or *toward* them. Previous research suggested that the more salient the distractor, the more pronounced is the curvature. We investigate the saliency of spatial visual features, predicted by a constrained maximum entropy model to be optimal or non-optimal information carriers in fast vision, by using them as distractors in a saccadic task. Their effect was compared to that of luminance-based control distractors. *Optimal* features evoke a larger saccadic curvature compared to *non-optimal* features, and the magnitude and direction of deviation change as a function of the delay between distractor and saccade onset. Effects were similar to those found with *high-luminance* versus *low-luminance* distractors. Therefore, model-predicted information optimal features interfere with target-oriented saccades, following a dynamic attraction–repulsion pattern. This suggests that the visuo-oculomotor system rapidly and automatically processes optimally informative features while programming visually guided eye movements.

## Highlights

• Optimally informative visual features interfere with the saccade path to a target
• Saliency determined by information maximization is comparable to luminance saliency
• Saccade curvature can be used as an objective measure of visual stimuli’s saliency

## Graphical abstract

**Figure:**
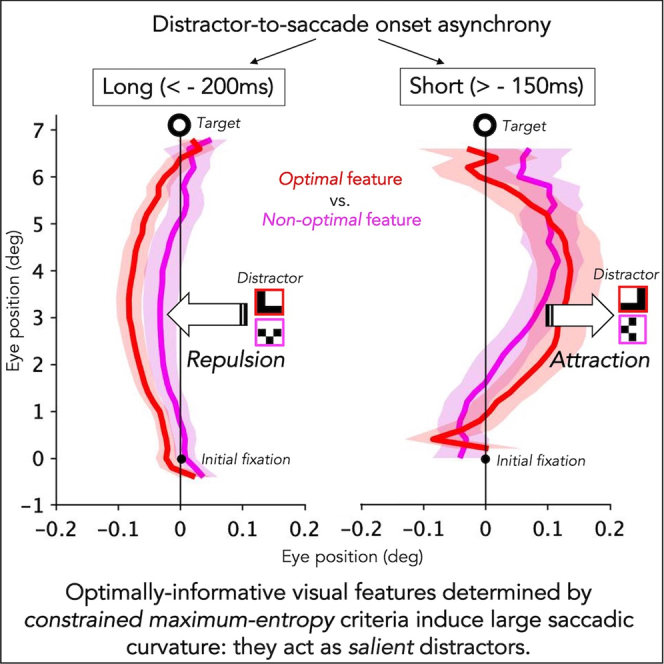

## Introduction

As demonstrated by classic studies on eye movements, the trajectory taken by the eye to reach a target position does not follow a straight line between the saccade starting and ending point.^1^^,^^2^ In recent years, a consistent number of studies have demonstrated that the magnitude and direction of this natural saccadic curvature can be modulated by the presence of a competing distractor stimulus presented with the saccade target. Indeed, visual distractors may cause a deviation either *toward* (attraction) or *away* from (repulsion) their location (for reviews see^3^^,^^4^).

Factors determining the direction of the curvature are still under investigation. Some studies suggested that the spatial distance between target and distractor modulates the curvature. For example, saccade trajectories tended to deviate toward the distractor location when this was presented close to the target, whereas trajectories deviated away from the distractor when presented closer to fixation.^5^ Differences also emerged based on target and distractor location predictability: when their location was unpredictable, trajectories deviated toward distractors, whereas, with predictable locations, saccades deviated away from distractors.^6^

Besides the role of spatial position, some studies have also shown that the temporal distance between distractor presentation and saccade onset influences its trajectory. When target and distractors were presented simultaneously, shorter-latency saccades (less than ∼200 ms) deviated toward distractors, whereas longer-latency saccades deviated away from distractors.^4^^,^^6^^,^^7^^,^^8^^,^^9^

Jonikatis and Belopolsky^10^ induced oculomotor competition by briefly presenting a task-irrelevant distractor (50 ms) at different times during the peri-saccadic epoch (from −400 to +600 ms from saccade onset). They found that the distractor offset time relative to saccade onset (DSOA) influenced the amplitude of the curvature; the deviation *away* was maximal when the distractor-to-saccade onset asynchrony was long and decreased as DSOA became shorter.^10^

Distractor stimuli can also influence saccades’ endpoint position. Indeed, when a distractor is presented in close spatial proximity to a target, saccades tend to land in between the two objects rather than on the target – the so-called “global effect”.^11^^,^^12^^,^^13^

Finally, the effects of a distractor are also reflected in the temporal properties of saccades. Indeed, distractors positioned at a remote location from the target evoke longer saccade latencies as compared to distractors close to a target (i.e., “remote distractor effect,” RDE^14^^,^^15^^,^^16^). Further studies also found that a distractor presented before the target reduces the saccadic latency, contrary to a distractor presented after the target which delays saccades latency,^17^ differently depending on whether it is in the same hemifield as the target and near or far from the fovea.^14^^,^^18^

It has been speculated that all these effects may be attributed to inhibitory processes occurring in the oculomotor system in situations where observers have to make a fast and accurate eye movement to a target while ignoring a competing distractor. Hence, the directional deviation of the saccade trajectory away or toward the distractor location would reflect the outcome of this competition. If the distractor location is only weakly inhibited the saccade trajectory will deviate toward the distractor before heading to the target,^3^^,^^19^^,^^20^^,^^21^ whereas strong inhibition will cause deviation away from the distractor.^20^^,^^21^^,^^22^^,^^23^^,^^24^

The fundamental point for our current study is that a larger saccade deviation either away or toward a distractor implies a stronger influence of the distractor (e.g., as obtained with larger or brighter distractors^12^^,^^25^). In other words, more salient distractors yield more pronounced competition, that in turn leads to stronger attraction and requires greater inhibition, inducing overall greater saccadic curvature. This interpretation has been broadly used to explain a variety of findings. For example, it has been speculated that distractors that share visual similarities with the saccade target produce greater trajectory deviation than dissimilar distractors because they are more behaviorally salient for the visuomotor system engaged in that particular saccadic task.^26^ A similar speculation based on a broad definition of saliency has been proposed for distractors closer to fixation versus distractors far from fixation,^5^ for bimodal distractors versus unimodal ones,^19^ and for abrupt onset versus color singleton distractors.^27^

Recent studies specifically tested distractor saliency effects on saccade curvature. Jonikaitis and Belopolsky^10^ used a double saccade task and manipulated the salience of the distractor presented before the first saccade by adjusting its luminance. They observed that the degree of curvature of the second saccade away from the distractor increased when the distractor’s luminance increased, suggesting that information about the distractor’s salience was also transferred across saccades.^10^ This saliency effect was later reproduced across different sensory modalities.^28^ Van Zoest et al.^29^ modified the distractor salience in terms of orientation contrast relative to the surrounding stimuli. Their results revealed that saccades deviated toward the irrelevant distractor and this deviation was stronger for more salient distractors, with a stronger effect on the shortest saccade latencies.^29^ Finally, Tudge et al.^30^ exploited the assumption of the relation between distractor saliency and saccade deviation to estimate the saliency of stimuli defined by combinations of different features with respect to single-feature stimuli. Many studies also found that distractors with task-relevant features produce deviations in saccade trajectories, showing that the visuo-oculomotor system is not just sensitive to low-level saliency, but also to high-level manipulations of distractors’ saliency.^31^^,^^32^^,^^33^^,^^34^^,^^35^ As an example, Hickey and van Zoest^36^ found that saccade trajectories are influenced by reward-associated distractors, demonstrating top-down, task-dependent influences on saccadic curvature.

Building up on these findings, in the present study, we compare the effects on saccades trajectories produced by different types of visual features used as distractors, considering the magnitude of curvature as a measure of feature saliency.

Here we adopt an operational definition of visual saliency, following the principle that it is proportional to the amount of local information^37^ available in the spatial arrangement of black and white pixels (*feature*) weighed by the system processing costs, as proposed by a recent constrained maximum entropy model of early visual feature extraction in fast vision.^38^ Fast vision processing is considered to occur within delays below 150 ms in higher visual areas,^39^ and as low as 25–50 ms for early visual areas processing.^40^

These authors provided a formal definition of saliency starting from a general problem faced by the visual system: the extraction of biologically relevant information from a large flux of input data in the shortest possible time,^41^ on which survival depends. In these conditions, given the system’s physiological limitations (because of energy costs of neural activity and the limited number of neurons^42^^,^^43^^,^^44^), an early and intensive data reduction must be operated,^45^^,^^46^^,^^47^ to create a compact representation of the visual scene – the so-called “*primal sketch*”^48^^,^^49^). However, in these previous studies, the features composing the *sketch* were based on a few simple primitives (edges, bars), and were defined *a priori* based on known properties of visual receptive fields and on the prominent edge detection ability of the visual system. Instead, the aforementioned approach considers computational limitations as a central element together with the information optimization principle to extract a number of visual features from the statistics of natural images, composing a compact saliency map of the visual scene.^38^ The focus on high-frequency features was set because their processing requires tighter computational limitations, given their prevalence in visual scenes.

Specifically, the reference model is based on some very general assumptions. First, the model assumes that, at an early stage, the reduction of the huge input data flow is achieved by filtering only those pieces of input data matching a reference set of features, disregarding any other information (*pattern matching*). Then, it considers the computing limitations of this early stage filter, assuming that there is only a fixed number of visual features that the system can recognize in input (*limited storage capacity*) and imposing a tight upper bound on the total amount of data that can be produced as output to be transmitted to the next stages of processing (*fixed output bandwidth*). Such a system is optimal from the point of view of delivering the maximum amount of information to the following processing stages (*maximum entropy output*), therefore the set of selected features must be information efficient. For a detailed explanation of the exact mathematical definitions of the model function, see.^38^

The authors then proposed that only *optimal* features are considered *salient* in fast vision and used to build a bottom-up saliency map, because they are those producing the largest amount of entropy allowed by the system constraints (i.e., they are optimal carriers of information). High-frequency features that do not fulfill the constrained maximum-entropy optimization criterion are considered *non-optimal* and should not be used to build the saliency map.

Model predictions were tested by applying it to a set of natural-scene photographs^50^ and using very small features. Of interest, the structure of the selected *optimal* features closely resembles the spatial structure of the well-known bar and edge-like receptive fields^51^ found in primary visual cortices. This similarity implies that these specific visual receptive fields represent the optimal way to transmit information in fast vision, and it represents evidence of the strong predictive power of this model. Indeed, differently to most computational models of early vision (e.g.,^52^^,^^53^^,^^54^^,^^55^), the reference model leads to the extraction of biologically plausible structures, to build bottom-up saliency maps, without assuming any known biological details. Therefore, the edge-detection functionality in itself seems to derive from very general principles of information maximization and computational limitations.^38^ On the other hand, *non-optimal* features discarded by the model have either a uniform luminance structure (features with large bandwidth occupation) or a “noisy” alternation of black and white pixels (features with large storage occupation).

In the original study,^38^ filtering natural images with *optimal* features provided highly compressed *sketches* of visual scenes which resulted in simplified yet informative transformations of the originals, akin to the concept of saliency maps. Indeed, optimal features turned out to be arranged along objects’ contours (edges and lines) rather than being scattered throughout the image. Human participants’ performance in discriminating original images based on very brief presentations (25 ms) of these sketches was very accurate, comparable to that obtained by using their grayscale original versions.^38^

In more recent experiments, we compared the relative saliency of optimal and non-optimal features with psychophysical and eye movement tasks, presenting the features in isolation (not arranged along contours) for a few milliseconds, without any clues coming from a global structure. We found that observers explicitly prefer *optimal* over *non-optimal* features even if presented in smaller numbers or at lower contrast,^56^ and that optimal features automatically attract covert and overt attention more than non-optimal features.^57^ Also, we found that local *optimal* features embedded in images’ fragments contribute to successful image reconstruction.^58^

Here, we further tested the bottom-up saliency predictions of the constrained maximum-entropy model by using optimal and non-optimal features as task-irrelevant distractors in a saccadic task and measuring the resulting saccadic curvature. This approach allowed us to study the low-level, automatic integration of these optimal features in fast visuo-oculomotor processes, along with its dynamics. We expected that if optimal features are indeed more salient, their presence will interfere in a quick and automatic manner with the ongoing oculomotor programming, and they will induce a larger saccadic curvature. As a control for this saliency effect, we compared it to the saccadic curvature induced by high-luminous versus low-luminous distractors. To characterize the time course of the saliency effect, we investigated changes in saccade trajectory deviations as a function of DSOA. We also looked at the possible effects of distractors’ saliency on endpoint position and saccade latency.

## Results

**Figure 1:**
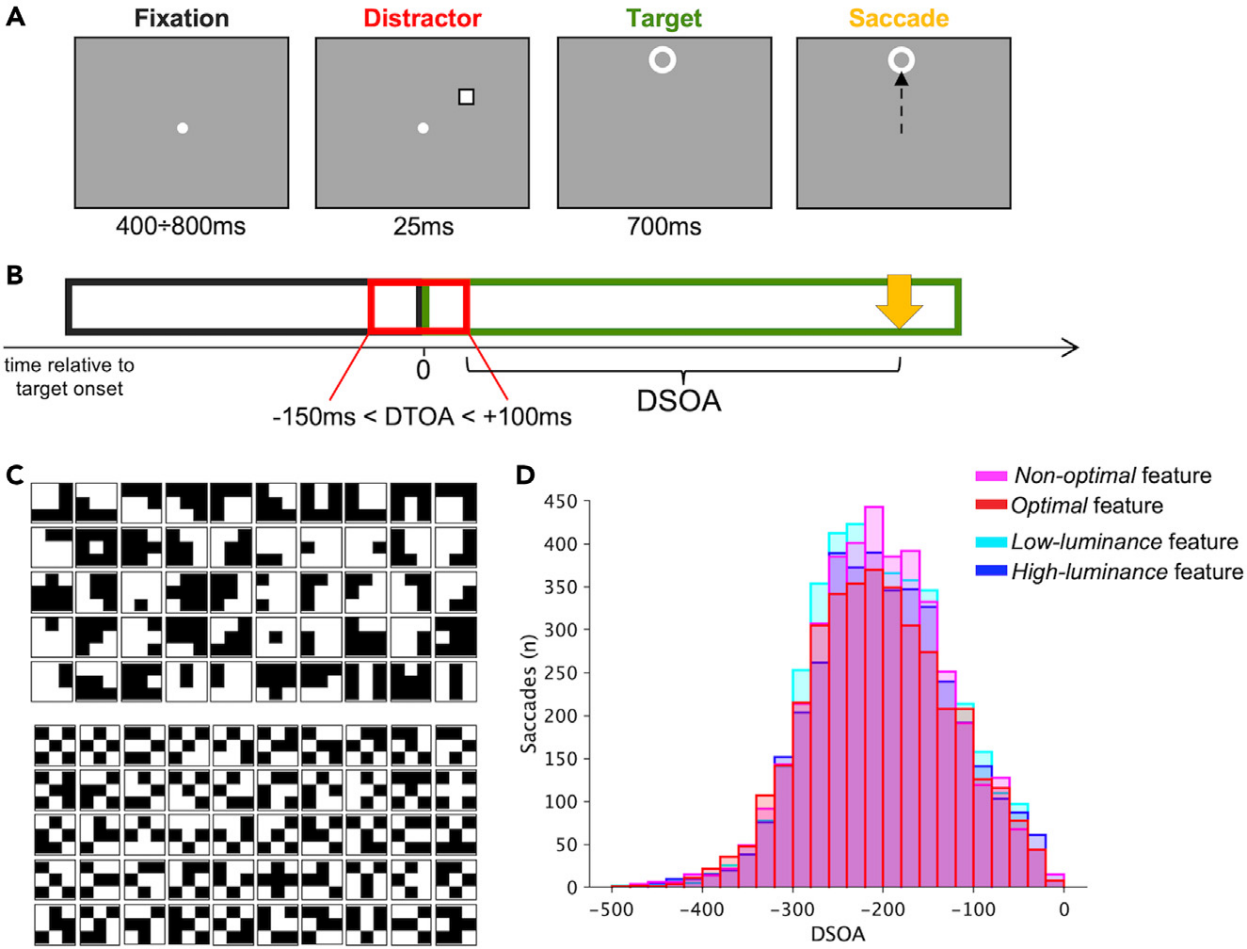
Procedure, stimuli, and DSOA distribution (A) Example of a trial. The duration of the different stimuli is reported under each panel. In this example, the distractor is presented as an empty square (9 by 9 pixels) and had a negative DTOA (presented before the target), and the target is presented 7° above the fixation. (B) Display sequence. The fixation point disappears as soon as the target appears. The time 0 refers to the target onset. Relative to target onset, the distractor could appear from 150 ms before the target to 100 ms after the target (DTOA = Distractor to Target onset asynchrony). The time distance between the distractor offset and the saccade onset (yellow arrow) is the distractor offset-to-saccade onset asynchrony (DSOA). (C) Distractor stimuli of the experimental condition—*optimal* and *non-optimal* features. Upper panel: Set of 50 *optimal* features considered as high salient features by the reference model.^38^ Lower panel: Set of 50 *non-optimal* features considered low salient features by the reference model.^38^ The features used here are those with the lowest probability in the statistical distribution of all possible 512 3 × 3-pixel black and white features. (D) DSOA distribution for each distractor condition. Data are binned every 20ms.

In the present experiment, observers were asked to perform a vertical saccade toward a visual target (7° above or below the fixation), whereas one brief task-irrelevant distractor could randomly appear on the right or on the left (25 ms), halfway from the target (for an example of a trial see Figure 1A). The distractor was randomly presented with a variable delay with respect to target onset (DTOA from −150 to +100 ms); the saccade toward the target could then start at different times from the distractor offset (DSOA) (Figure 1B). The distractor stimulus in the experimental condition could be an optimal feature (deemed high salient distractor; Figure 1C**—upper panel**) or a non-optimal feature (deemed low salient distractor; Figure 1C**—lower panel**); in the luminance-control condition, the distractor could be a *high-luminance* (high salient) or a *low-luminance* (low salient) feature. In some trials, no distractor was presented, and this condition was used as a baseline. For each normalized saccade (see STAR Methods for the definition), we measured its trajectory, curvature, endpoint, and latency. See STAR Methods for more details on the task, the stimuli, and the eye movement analysis.

Here, our interest is measuring the effect of a distractor on the saccade trajectory assuming that its representation will be further inhibited with a longer delay between the distractor and the saccade. For this reason, we considered the temporal distance between the distractor presentation and the saccade onset (DSOA) as the relevant variable for the direction of the saccade’s deviation. Previous works on the effect of a distractor on saccadic trajectory have considered the saccadic latency as the independent variable because the target and the distractor were usually presented simultaneously, so latency and DSOA were the same.^3^^,^^4^^,^^6^^,^^9^^,^^29^ Saccade distribution for each distractor type across the DSOA time course (bins of 20ms) is reported in Figure 1D. In the following analyses, we considered the DSOA values between −340ms and −20ms, the interval in which there are a reasonable number of saccades to reconstruct the time course (20-ms bins including at least 40 saccades for each distractor condition).

**Figure 2:**
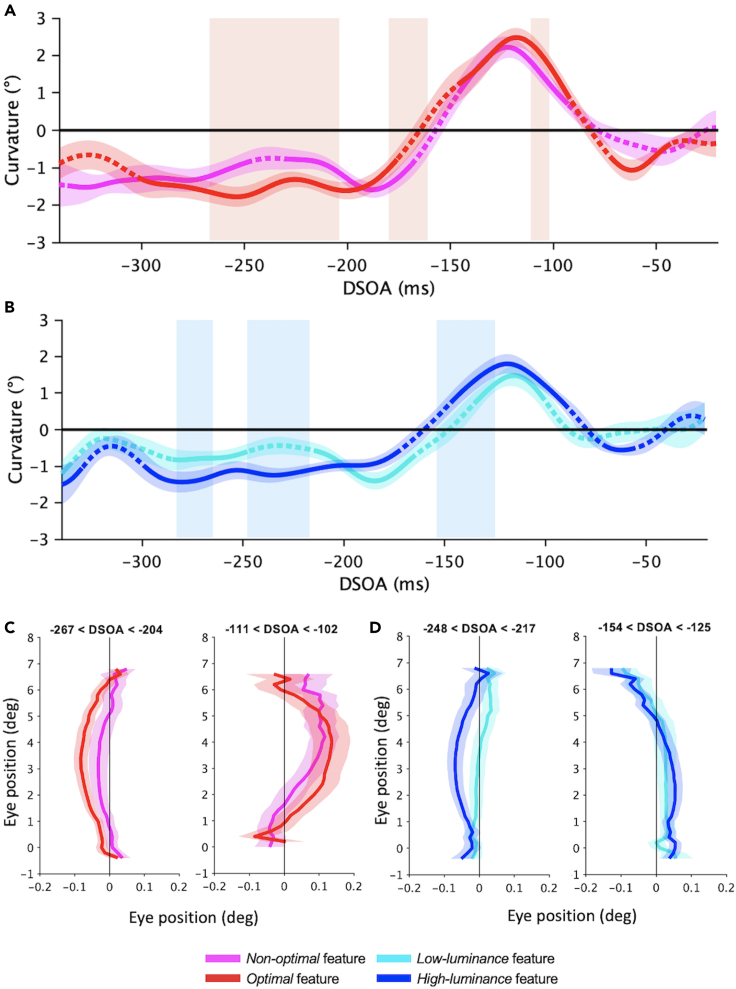
Saccadic curvature (A and B) Curvature time course as a function of DSOA (SMART analysis^59^) for *optimal* (red) versus *non-optimal* (pink) features (A), and for high-luminance (blue) versus low-luminance (cyan) features (B). Shaded areas around the curves: 95% confidence interval. Colorful shaded rectangles: time intervals with significantly different curvature induced by high-salient versus low-salient distractors (p < 0.01; light-red: *optimal* versus *non-optimal* features; light-blue: high versus low-luminance features). Solid parts of lines: time windows (ms) with curvature significantly different from zero (p < 0.01; baseline versus *non-optimal* features: [−336, −247] ms, [−229, −169] ms, [−146, −93] ms; baseline versus *optimal* features: [−302, −176] ms, [−145, −92] ms, [−72, −43] ms; baseline versus *low-luminance* features: [−340, −336] ms, [−285, −248] ms, [−214, −166] ms, [−134, −102] ms; baseline versus *high-luminance* features: [−340, −33] ms, [−298, −174] ms, [−147, −90] ms, [−70, −57] ms). Dashed parts of lines: curvature compatible with zero. (C) Experimental condition - Average trajectories. Left panel: saccades with DSOA between −267 ms and −204 ms showing deviation away from the distractor; right panel: saccades with DSOA between −111 ms and −102 ms showing deviation toward the distractor. (D) Luminance-control condition - Average trajectories. Left panel: saccades with DSOA between −248 ms and −217 ms showing deviation away from the distractor; right panel: saccades with DSOA between −154 ms and −125 ms showing deviation toward the distractor. Errors are SE across participants.

First, we analyzed saccadic curvature as a function of the DSOA with the SMART procedure (Figure 2), a smoothing method designed for the analysis of response time courses that retains high temporal resolution with no need to define time bins^59^ (see quantification and statistical analysis section in the STAR Methods). All saccades’ trajectories shown in the following graphs are flipped so that the target is always in the upper field and the distractor is always on the right; namely, negative curvature indicates deviation away from the distractor (“repulsion”), whereas positive curvature indicates deviation toward the distractor (“attraction”).

Saccades time courses for *non-optimal* versus *optimal* features, and for *low-luminance* versus *high-luminance* features, are reported in Figures 2A and 2B, respectively. Common across all conditions is that, at large negative DSOA, the presentation of all distractors induced a negative deviation, significantly different from the natural deviation measured in the condition without a distractor (baseline condition). Around DSOA of −200 ms the deviation away decreases, until becoming significantly positive after −150 ms. After reaching the peak around −120 ms, the curvature begins to decrease. After −100 ms, the curvature becomes compatible with zero for the low-salient distractors (*non-optimal* and *low-luminance* features) and slightly negative for the high-salient distractors (*optimal* and *high-luminance* features). All significant DSOA time windows between all types of distractors conditions versus the no-distractor baseline condition are reported in the caption of Figure 2.

Time courses for each distractor condition have been compared with each other. For each comparison, we report the time window of the significant cluster (ms), the average of smoothed Gaussian data, and the 95% confidence interval within each significant cluster (p < 0.01).

Comparing the curvature induced by the two types of distractors of the experimental condition (Figure 2A), we observed that, at large negative DSOA ([−267, −204] ms), *optimal* features induce a larger deviation *away* from their location than that induced by *non-optimal* features (−1.55° versus −0.89° ± 0.2°). At intermediate DSOA ([−180, −161] ms*) optimal* features induce less deviation away than *non-optimal* (−0.39° versus −0.97° ± 0.2°) and, finally, at short DSOA ([−111, −102] ms) they produce a larger deviation *toward* their position than that induced by *non-optimal* features (2.05° versus 1.54° ± 0.2°).

Similar results emerged in the luminance-control condition (Figure 2B). Indeed, at long DSOA ([−283, −265] ms and [−248, −217] ms), *high-luminance* distractors induce a stronger repulsion than that induced by *low-luminance* distractors (−1.38° versus −0.8° ± 0.1 and −1.19° versus −0.49° ± 0.2°), whereas, at shorter DSOA ([−154, −125] ms), *high-luminance* distractors induce a stronger attraction than *low-luminance* distractors (1.10° versus 0.48° ± 0.2°).

Overall, these results indicate that high-salient features, such as very bright features as well as optimally informative features, induce a larger curvature in the saccade trajectory than that induced by lower salient distractors (less bright or less optimal), and this holds both when the curvature is toward and away from the distractor.

Some differences also emerged by comparing the curvature induced by distractors of the experimental versus control conditions (not reported in the figure). Indeed, at long DSOA, the deviation *away* is larger for *non-optimal* versus *low-luminance* features ([−324, −303] ms: −1.4° versus −0.33° ± 0.3°), as well as for *optimal* versus *high-luminance* features ([−263, −245] ms: −1.8° versus −1.1° ± 0.23°). Also, at short DSOA, the deviation *toward* is larger for *non-optimal* versus *low-luminance* features ([−138, −121] ms: −2° versus 2° ± 0.3°), as for *optimal* versus *high-luminance* features ([−127, −118] ms: −2.3° versus 1.8° ± 0.3°). Therefore, both repulsion and attraction effects are more pronounced in the presence of experimental distractors than uniform-luminance distractors.

Examples of average saccades trajectories in the trials with *non-optimal* versus *optimal* features, and *low-luminance* versus *high-luminance* features, within two significant time windows found with the SMART procedure, are reported in Figures 2C and 2D, respectively. Data shown in panels C and D are saccades trajectory averaged across participants with shaded areas representing the standard error of the mean (SE; see STAR Methods for details).

Additional analyses show that curvature effects of *optimal* versus *non-optimal* features cannot be explained by their spatial frequency (see Figures S1–S3).

**Figure 3:**
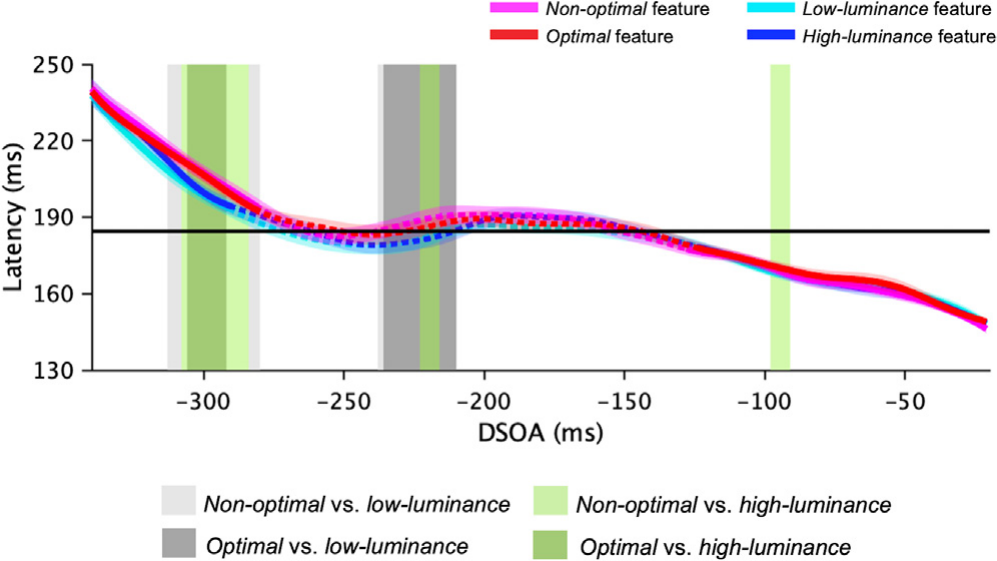
Latency Latency time course as a function of DSOA (SMART analysis^59^) for *optimal* features (red), *non-optimal* features (pink), high-luminance features (blue), and low-luminance features (cyan). Shaded areas around the curves: 95% confidence intervals. Solid parts of lines: time windows (ms) with latency significantly different from baseline (p < 0.01; baseline versus *non-optimal* features: [−340, −281] ms, [−128, −21] ms; baseline versus *optimal* features: [−340, −280] ms, [−124, −21] ms; baseline versus *low-luminance* features: [−340, −291] ms, [−122, −21] ms; baseline versus *high-luminance* features: [−340, −290] ms, [−124, −21] ms). Dashed parts of lines: latency compatible with baseline. Colorful shaded rectangles: time intervals with significantly different latency with experimental versus luminance-control distractors (color legend in the figure).

The latency of saccades was also analyzed as a function of DSOA (from −340 ms to −20 ms) with the SMART procedure (Figure 3). The average latency of saccades in the trials with no distractor is 184.5 ± 2.8 ms (SEM across participants) (horizontal black line in Figure 3). Common across all trials with a distractor is that the saccadic latency is longer at the longest DSOA (<−250 ms), and shorter at the shortest DSOA (>−250 ms) compared to the baseline latency. All significant DSOA time windows between all types of distractor conditions versus the no-distractor baseline condition are reported in the caption of Figure 3.

No significant differences were detected when comparing the latency time courses obtained with high-salient versus low-salient distractors in both experimental and control conditions. Instead, some differences emerged between the two conditions. Indeed, there are some time windows (ms) where the latency (ms) for low-luminance distractors is shorter than for *non-optimal* features ([−313, −280] ms: 197 versus 205 ± 2 ms; [−238, −214] ms: 181 versus 188 ± 2 ms) and *optimal* features ([−308, −284] ms: 196 versus 203 ± 2 ms; [−98, −91] ms: 167 versus 171 ± 1 ms). In the same way, with saccade preparation for high-luminance distractors is more delayed than for *non-optimal* features ([−300, −294] ms: 197 versus 205 ± 2 ms; [−236, −210] ms: 181 versus 188 ± 1 ms) and *optimal* features ([−306, −292] ms: 199 versus 205 ± 1 ms; [−223, −216] ms: 181 versus 186 ± 1 ms). This result suggests that saccadic latency is not sensitive to the degree of saliency (either when considering differences in luminance or in amount of information), rather it seems to be influenced by other distractors characteristics. See the discussion section for possible explanations of this result.

**Figure 4:**
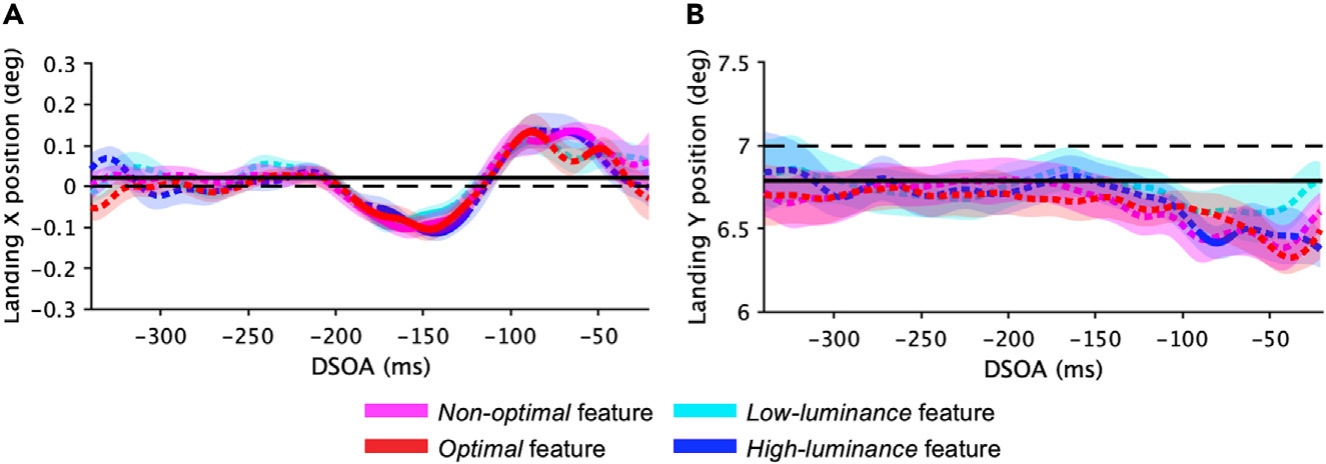
Landing point Landing X- (A) and Y-position (B) time course as a function of DSOA (SMART analysis^59^) for *optimal* features (red), *non-optimal* features (pink), high-luminance features (blue), and low-luminance features (cyan). Shaded areas: 95% confidence interval. Solid parts of lines: time windows (ms) with landing X- and Y-positions significantly different from baseline (p < 0.01). (A) baseline versus *non-optimal* features: [−87, −132] ms, [−86, −54] ms; baseline versus *optimal* features: [−191, −123] ms, [−100, −79] ms, [−58, −45] ms; baseline versus *low-luminance* features: [−187, −132] ms, [−101, −89] ms; baseline versus *high-luminance* features: [−174, −125] ms, [−93, −85] ms, [−70, −57] ms. (B) baseline versus *high-luminance* features: [−91, −72] ms. Dashed parts of lines: landing X- and Y-positions compatible with baseline.

Finally, the landing point of saccades was also analyzed as a function of DSOA (from −340 ms to −20 ms) with the SMART procedure (Figure 4). Saccades in trials with no distractor, and a target positioned at 7° upward, on the vertical meridian, land on average at 0.2° ± 0.1° on the x axis (black horizontal solid line in Figure 4A), and at 6.8° ± 0.13° on the y axis (black horizontal solid line in Figure 4B). Thus, when performing a saccade toward the target, the baseline landing point is in a position slightly lower right than the actual target position (dashed horizontal black lines in Figures 4A and 4B). Landing x-position of saccades for any type of distractor is compatible with the baseline position until around −200 ms. Then, we observed a significant shift in the opposite direction of the distractors (negative landing X-point) for about 100 ms, and finally a shift toward the distractor (positive landing X-point) between −100 ms and −50 ms (Figure 4A). Landing y-position for all types of distractors is compatible with the baseline position across almost all DSOA times, with a small tendency of shifting downward (below the target) at the shortest DSOA (significant only for the high-luminance distractor in a very small time-window; Figure 4B). All significant DSOA time windows between all types of distractors conditions versus the no-distractor baseline condition are reported in the caption of Figure 4.

Comparing the landing point time courses obtained with high-salient versus low-salient distractors in both experimental and control conditions, we could observe that the landing position does not change with distractor saliency; neither in the horizontal (Figure 4A) nor in the vertical dimension (Figure 4B). Also, no differences emerged between experimental and control distractors.

We finally analyzed starting X- and Y-positions of saccades (not shown in the figure), finding that they do not differ across distractor conditions nor with DSOA. They are always compatible with the average baseline starting positions (x = −0.01° ± 0.1°, y = 0.1° ± 0.02°) found in the no-distractor condition.

## Discussion

In the present study, we compared the saliency of some local features, originally identified as optimal or non-optimal information carriers based on constrained-entropy maximization criteria.^38^ To do this, we presented these features as distractors in a simple saccadic task and used the saccadic curvature as a measure of their relative saliency. Our main objective was to investigate whether the saliency provided by features’ optimality, which is already known to induce an automatic attentional attraction,^56^^,^^57^ also occurs so rapidly that it can influence the trajectory of a planned saccade. For our main goal, the saccadic curvature has been merely used as a means to quantify the model-predicted visual saliency. However, this study may also serve to better characterize the temporal factors modulating the saccadic curvature and shed some light on the saccadic programming dynamics in the presence of distracting stimuli.

Our main result is that the saccadic curvature induced by *optimal* features is larger than that produced by *non-optimal* ones, suggesting that *optimal* features act as highly salient distractors which strongly compete with the target location in the oculomotor centers. The results obtained in the luminance-control condition, in which we compared the deviation induced by distractors with high or low luminance contrast, also support our main hypothesis. Indeed, high-luminance features induce a larger deviation than low-luminance features, and the amplitude of the deviation is comparable to that found with *optimal* features. This suggests that the interference on saccade programming produced by differences in features’ optimality is as powerful as that produced by luminance-based saliency. Note that all types of distractors, also *non-optimal* and *low-luminance* features, evoke some degree of curvature compared to the condition where no distractor is presented. This confirms the general finding that the path of target-oriented saccades is influenced by visual competing distractors even if they are task-irrelevant and low visually salient (for reviews see.^3^^,^^4^

Interesting results also emerged by the inspection of the time courses of saccade trajectory deviations. Indeed, we found that the saccadic curvature direction changes as a function of the temporal distance between the saccade onset and the distractor offset. In both experimental and luminance-control conditions, saccades starting at least 200/150 ms after the distractor tend to deviate away from it, whereas when the temporal interval between distractor offset and saccade onset is small saccades curve toward the distractor location. When comparing the saliency effect within the experimental and within the control condition, one can observe a small relative shift of the time intervals with a significantly different curvature for the more salient stimuli as compared to the less salient ones. For instance, *optimal* features induce a significantly larger curvature toward the distractor, compared to *non-optimal* features already for DSOAs around −100ms, whereas the difference between high-luminance and low-luminance distractors becomes significant only if the distractor is presented more than 125ms before saccade onset. Yet, the qualitative agreement of the time course of saliency effect across experimental and control conditions is apparent. Further studies will be needed to precisely investigate possible quantitative differences between optimal feature-based and luminance-based saliency in more detail.

When considering the relationship between curvature and saccadic latency, our results confirm previous findings, showing a trend for long latency saccades to have larger deviations away from distractors than fast saccades.^4^^,^^8^^,^^9^

Results also show that our experimental distractors, both *optimal* and *non-optimal*, evoke larger deviations than those induced by our control uniform-luminance distractors. Similarly, saccadic latency increases more with optimal and non-optimal features than with uniform-luminance distractors. There are some possible explanations for these results, which would deserve further investigation. First, our experimental stimuli have an internal structure, not present in the control stimuli. Thus, we could speculate that these results are related to recent findings about saccade-contingent neuronal activity in the superior colliculus being tuned to the spatial frequencies of a visual stimulus.^60^ In addition, our findings could also be explained by experimental features’ internal contrast. Indeed, optimal and non-optimal features, although having the same average luminance contrast with respect to the background as the control features (Weber contrast), they have a higher internal luminance contrast compared to them (RMS contrast). Finally, because optimal and non-optimal distractors are more complex than the simple uniform-luminance distractors, the perceptual load needed for their analysis could be higher than that required by the control stimuli,^61^ thus influencing saccadic curvature and latency.

More in general, in our study, we found an increase of saccadic latency with all distractors at long DSOA. This is in line with previous studies, showing that the presentation of a competing stimulus with the target delays saccade onset (RDEs^6^). However, we did not find an increase nor a decrease in saccadic latency in the presence of a highly salient distractor compared to low salient distractors. The absence of any interference in the temporal domain may be the result of the low uncertainty about target locations: the target was always presented directly above or below fixation and so participants were potentially able to prepare an eye movement in advance to these two relevant target locations.^19^

We did not find any differences in saccades’ endpoint positions across different distractor types and saliency levels. This result could be somehow unexpected because prior research found distractor-saliency effects on endpoint deviations (e.g.,^31^^,^^33^^,^^35^). The discrepancy between our results and the previous ones may be related to the fact that shifts in saccades ending points toward the distractor are usually found in specific conditions of spatial proximity between target and distractor (Global effect^11^^,^^12^^,^^13^), and high uncertainty about their position.^6^^,^^62^ In our paradigm, these factors were not optimized for observing the Global effect, and this might explain why the saccadic landing point does not change with the distractor condition. When analyzing the landing point time course as a function of DSOA, we found that the landing point of saccades starting way after the distractor is compatible with the natural landing point of saccades when no distractor is presented. Instead, saccades starting shortly after the distractor offset landed in a position shifted from the baseline. We can speculate that the effect on the saccadic landing point could be simply a sort of automatic “over correction” after the initial deviation caused by the distractor.

On a more general ground, distractor effects have been explained in terms of the programming of an oculomotor vector toward the target and distractor locations. The oculomotor system has to resolve the competition between these two vectors to determine the goal of the next eye movement.^63^^,^^64^ In this process, the vector programmed toward the distractor needs to be inhibited to avoid making an eye movement toward it rather than toward the instructed target.^65^^,^^66^ The intermediate layers of the superior colliculus (SC) are often implicated in oculomotor competition^67^^,^^68^^,^^69^^,^^70^^,^^71^ because they contain a large population of neurons with large overlapping *motor fields* that encode the saccadic displacement^72^^,^^73^ which can be regarded as forming a “motor map”. When the population of neurons encoding the target overlaps with a second population encoding the distractor, an error in the computation of the initial saccade direction may occur. The initial saccade deviation, either toward or away from a competing distractor, is thought to reflect the level of neural activity at the distractor site around the time of saccade onset.^74^ Therefore, when the competition is unresolved and there is strong activity at the distractor site, the saccade will deviate toward its location,^6^ coherently with our results obtained when saccades started close in time to the distractor. In contrast, later in time, when the inhibition of distractor-related activity is achieved, saccades can even deviate away from it,^27^ as we found for saccades starting way after the distractor offset. It has been assumed that the stronger the inhibition required by the distractor, the larger the deviation away.^65^^,^^66^ This would explain why our high salient distractors induce a larger deviation away.

Although spatiotemporal factors that relate local excitation at the distractor site in the SC to saccades curved toward distractors are well understood,^74^^,^^75^ the mechanisms responsible for saccades curved away from distractors are more controversial^76^^,^^77^ (see also^78^^,^^79^ for related models).

Recently, on the basis of behavioral measurements, Kehoe and Fallah^80^ proposed a model of activation and inhibition explaining both toward and away curvature. In particular, they modeled DSOA functions separately for saccades curved toward and away from distractors and suggested that a similar temporal process determined the magnitude of saccade curvatures in both contexts. Their behavioral and theoretical results are in line with our findings.^80^

To summarize, our work extends and generalizes the notion that the saccadic curvature toward or away from a distractor increases with the saliency of the distractor presented in competition with the saccade target. In fact, we show that the relative saliency of optimal versus non-optimal features predicted by the constrained maximum entropy model is reflected in the magnitude of the saccadic curvature. Our findings also indicate that the saliency based on information maximization has a comparable effect to luminance-based saliency and a similar time course. We conclude that the automatic capture exerted by optimally informative visual features results from early processes in the visual stream, such that it can interfere with the correct programming of fast, visually guided saccades. Finally, our study confirms that saccadic curvature can be used as an objective measure to quantify the relative saliency of a visual stimulus.

### Limitations of the study

In our experiment, the distractor was always presented at the same spatial distance between the fixation and the target, in a relatively well-predictable configuration. We think that by manipulating the spatial configuration, some previously observed distractor-saliency effects on saccadic latency and landing point not found here could emerge as well, potentially contributing to a complete picture of the interference mechanisms at play.

## STAR★Methods

### Key resources table

**Table:** 

REAGENT or RESOURCE	SOURCE	IDENTIFIER
**Deposited data**		

Raw and analyzed eye tracking data	This paper	Zenodo database: https://doi.org/10.5281/zenodo.7296587

**Software and algorithms**		

MATLAB R2018b	Mathworks, Natick, MA, USA	http://www.mathworks.com/products/matlab/
Psychtoolbox 3, including Eyelinktoolbox	Psychtoolbox.org	http://psychtoolbox.org/
JASP version 0.11.1	JASP Team	https://jasp-stats.org/

**Other**		

Eyelink 1000 eye tracker	SR Research Ltd., Ontario, Canada	http://www.sr-research.com

### Resource availability

#### Lead contact

Further information and requests for resources should be directed to and will be fulfilled by the lead contact, Serena Castellotti (serena.castellotti@unifi.it).

#### Materials availability

This study did not generate new unique reagents.

### Experimental model and study participant details

Twenty-three healthy volunteers participated in the present study (aged 22–34 years, M = 26.48, SD =3.07, fourteen females and nine males, all Caucasian). All observers had a normal or corrected-to-normal vision, provided written informed consent prior to their inclusion in the study, and were naive to the purpose of the experiment. The *Comité de Protection de la Personne (CPP*: *18*.*11*.*07*.*69231*) approved the experimental paradigm, which complied with the Declaration of Helsinki.

### Method details

#### Setup

Each participant was tested individually in a dark room. Stimuli presentation was controlled by a MacPro computer (OS 10.6.8), using the Psychophysics Toolbox^81^^,^^82^^,^^83^ and the Eyelink Toolbox extensions^84^ for Matlab. The experiment was displayed on a 120 Hz CRS Display++ LCD monitor (1920 by 1080 pixel), subtending 70 by 40 degrees at a viewing distance of 57 cm. Participants’ viewing was binocular, but only the right eye was recorded by an Eyelink 1000 video-based eye tracker (1 kHz). The observers’ head was stabilized with a chin—and forehead rest.

#### Procedure

At the beginning of the experimental session, a standard nine-point gaze-calibration routine was run and possibly repeated to ensure good quality of the eye movements recordings. The whole task (and the calibration) was presented on a grey display background (16 cd/m^2^; greyscale value = 127). See Figure 1A for an illustration of a trial. Each trial started with the presentation of a white central fixation point (44 cd/m^2^) for a variable duration, uniformly distributed in the range between 400–800 ms, followed by the target (white ring, 18 pixels large, 0.6°) shown for 700 ms at 7° eccentricity on the vertical meridian, above or below the initial fixation. A small distractor stimulus (arrays of 9 × 9 pixels, 0.3°, see stimuli section for details) could randomly appear for 25 ms on the right or on the left, halfway from the target (3.5° vertically and horizontally from fixation), with a variable delay with respect to target onset (Distractor-to-Target Onset Asynchrony—DTOA from -150 to +50 ms). See Figure 1B for an illustration of the display sequence. Observers were instructed to make a fast and accurate saccade toward the target. The following trial started only after participants resumed fixation. Each participant performed 1000 trials in total, in a single session with a small pause every 100 trials.

Task characteristics were chosen to maximize the possibility of observing curvature effects. First, we instructed only vertical saccadic eye movements because curvature effects are more pronounced for vertical than for horizontal saccades.^4^^,^^10^ Second, the target could only appear at two possible locations, because it has been found that predictable target locations make inhibitory mechanisms more pronounced in the target selection process.^6^^,^^21^ Also, utilizing different DTOA produce saccades starting at different times to the distractor (distractor offset-to-saccade onset asynchrony—DSOA) and allows us to analyze the temporal dynamics of the distractor’s effects on the saccade programming.

#### Stimuli and conditions

Trials were distributed across three different conditions: baseline (200 trials), experimental (400 trials), and luminance-control (400 trials) condition. Trials’ order was randomized. In the baseline condition, no distractor was presented. The experimental and luminance-control conditions differ for the visual distractor stimuli used in the task: in each condition, the distractor could be high salient (200 trials) or low salient (200 trials). In the experimental condition, distractors’ saliency differs based on the spatial arrangement of their black (greyscale value = 50; luminance 4 cd/m^2^) and white (greyscale value = 250; 44 cd/m^2^) pixels, whereas, in the control condition, distractor saliency was based on their luminance contrast with respect to the background.

Specifically, in the experimental condition, two types of distractors were used: *optimal* features and *non-optimal* features, as identified by the reference constrained maximum-entropy model^38^ (see the introduction section). At each trial, the *optimal* feature, assumed to be a “high salient distractor,” was randomly extracted from a set of 50 features selected as the best information carriers (Figure 1C**—Upper panel**; average greyscale value = 151; Weber contrast = 0.19; RMS contrast: M = 1.2, SD = 0.1, range [0.98 1.3]); whereas the *non-optimal* feature, used as a “low salient distractor,” was extracted from a set of 50 features selected amongst those with the lowest probability of occurrence in the statistical distribution of all possible features (Figure 1C**—Lower panel**; average greyscale value = 149, Weber contrast = 0.18; RMS contrast: M = 1.2, SD = 0.05, range [1.0 1.3]), and thus classified as poorly informative by the model. On average, then *optimal* and *non-optimal* features do not differ in their contrast properties. We also assessed the spatial frequency content (SF) of *optimal* and *non-optimal* features. The theoretical range of the unidimensional spatial frequency for our 9 x 9 pixels features is between 1.67 and 5 cycles/deg for both sets. In addition, to compare the spatial frequency content of our set of features we computed the distance of the spatial frequency (SF) spectra within and between our two sets of stimuli. This distance is defined as the Euclidian distance of the 9-dimensional vectors of Fourier components’ amplitude, each being a 3x3 two-dimensional spectrum (see Figures S1–S3).

These specific sets of *optimal* and *non-optimal* features have been already used in our previous works, in which we demonstrated that they are explicitly considered different for their saliency^56^ and that they have a different attention-grabbing power.^57^

In the luminance-control condition, the distractor stimulus could be a grey uniform square with low luminance contrast with respect to the background (20 cd/m^2^; greyscale value = 150; Weber/RMS contrast = 0.18; SD = 0), or a white uniform square with high luminance contrast (23 cd/m^2^; greyscale value = 173; Weber/RMS contrast = 0.36, SD = 0). The luminance values of the control distractor stimuli were purposely chosen to compare their effect with the effect induced by the distractor used in the experimental condition. Indeed, these values produce a contrast corresponding to the “equivalent contrast” found to match the saliency difference between *optimal* and *non-optimal* features.^56^

#### Data processing

Data about saccades were stored and analyzed offline with MATLAB routines (The MathWorks, Inc.). Recorded horizontal and vertical gaze positions were low-pass filtered with a Butterworth (acausal) filter of order 2 with a 30 Hz cutoff frequency and then numerically differentiated to obtain velocity measurements. An automatic conjoint acceleration and velocity threshold method was used to detect saccades.^85^

For each trial, the first saccade after target onset was analyzed. Trials without recorded saccades (e.g., due to a long blink), saccades starting further than 2° from the fixation point, saccades landing further than 2° from the target, and saccades with latency lower than 80 ms or longer than 500 ms (with respect to target onset), were excluded (∼15% of all saccades).

For each correct saccade, we extracted its latency, starting point, landing position, and curvature. To obtain the latter, we first smoothened the gaze position trajectory by computing the mean horizontal position for each 0.2° spatial bin on the vertical axis. By doing this, a unique pair (x_i,_ y_i_) was obtained for each bin between the y-position of the fixation and of the target location, removing a possible ambiguity in the definition of the saccadic curvature. Then, we normalized each trace by subtracting the mean trajectory (horizontal and vertical eye position) obtained in the trials without distractors (no-distractor baseline condition), separately for upward and downward saccades. This normalization ensured that any deviation of the saccade trajectory in response to a distractor was not due to the idiosyncratic curvature of the saccade trajectory of individual participants. Normalized y-coordinates were rotated to direct all saccades upward and, finally, normalized x-coordinates were inverted for trials in which distractors were presented counterclockwise (i.e., to the left) relatively to the target direction. This way, positive and negative values represent coordinates and curvature angles that were directed either toward or away from the distractor’s head-centered position, respectively. With these coordinates we then determined the saccade curvature angle for each trial, that is, the median of the angular deviations of each sample gaze-position point from a straight line connecting the starting and ending point of the saccade.

### Quantification and statistical analysis

For each saccade, we calculated the DSOA, which is the distractor offset-to-saccade onset asynchrony in milliseconds.

The time course of saccadic curvature, latency, landing point, and starting position as a function of the DSOA has been analyzed with the SMART procedure, a smoothing method for the analysis of response time courses.^59^ Data were analyzed at a 1-ms resolution and smoothed with a Gaussian kernel of 10 ms width. 1000 permutations were run for every test. We set the significance level as *p* < 0.01. Time courses for each distractor condition have been compared with each other, and each condition has been compared with the baseline condition. Data have been compared in the DSOA window between -340 ms and -20 ms. This interval has been chosen by looking at the distribution of DSOA across all trials (Figure 1D) and selecting the 20 ms bins which included at least 40 saccades for each distractor condition. For each comparison, we report the time window of the significant cluster, the average of smoothed Gaussian data and 95% confidence interval within each significant cluster.

To produce figures of average saccade trajectories for each distractor condition (Figures 2C and 2D), we averaged the normalized trajectories of all participants and estimated their standard error of the mean (SEM). Since each participant’s trajectory may have different starting/ending point (therefore different lengths in the y-coordinates), only y-coordinates including at least fifteen participants (i.e., fifteen x-coordinates) were considered. This process led to final average trajectories having the central part populated by all participants, and the outermost parts containing only some participants (with a minimum of 15 participants).

## Data Availability

• Eye tracking and behavioral data have been deposited at Zenodo and are publicly available as of the date of publication. Accession numbers are listed in the key resources table. • This paper does not report original code. • Any additional information required to reanalyze the data reported in this paper is available from the lead contact upon request.
