# Supplementary material for: Saccadic trajectories deviate toward or away from optimally informative visual features
Source: iScience. 2023 Jul 4;26(8):107282. doi: 10.1016/j.isci.2023.107282 (PMC10371840; doi:10.1016/j.isci.2023.107282)
Supplement: Document S1. Figures S1–S3 [file mmc1.pdf]

iScience, Volume *26*

Supplemental information

Saccadic trajectories deviate toward or away from optimally informative visual features

Serena Castellotti, Martin Szinte, Maria Michela Del Viva, and Anna Montagnini

SUPPLEMENTAL INFORMATION

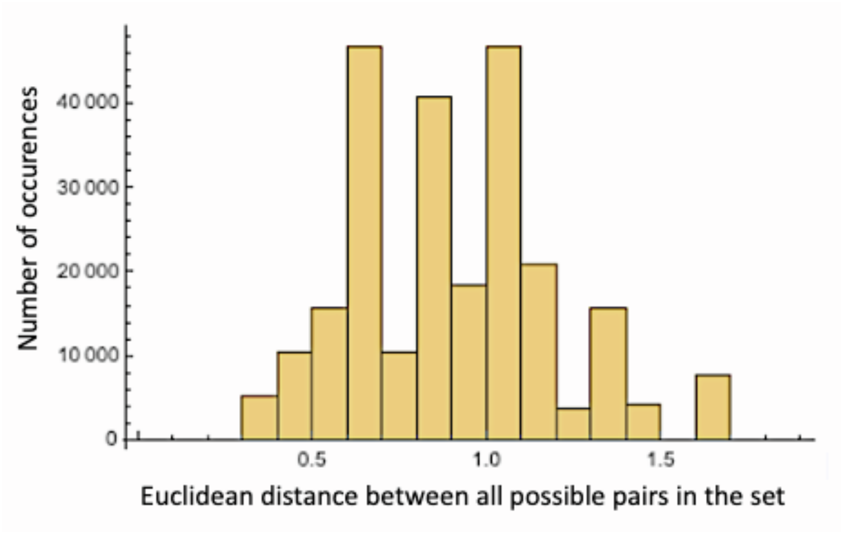

Figure S1. Distances within the set of all possible features, related to STAR Methods. To compare the spatial frequency content of our set of features, the distance of the spatial frequency (SF) spectra (defined as the Euclidian distance of the vectors of Fourier components' amplitude) within and between our two sets of stimuli has been calculated. The spectra have been computed after subtracting the mean value from each feature, to remove the irrelevant constant component. The spread of frequency spectra within a given set of features is visualized by plotting a histogram of the above-defined distances, taken between all possible pairs within the set. The histogram of distances within the set of all possible 512 3x3 binary features (excluding degenerate cases with distance < 10-4, due to symmetrical/negative features) shows that they are comprised in the range [0.3, 1.7].

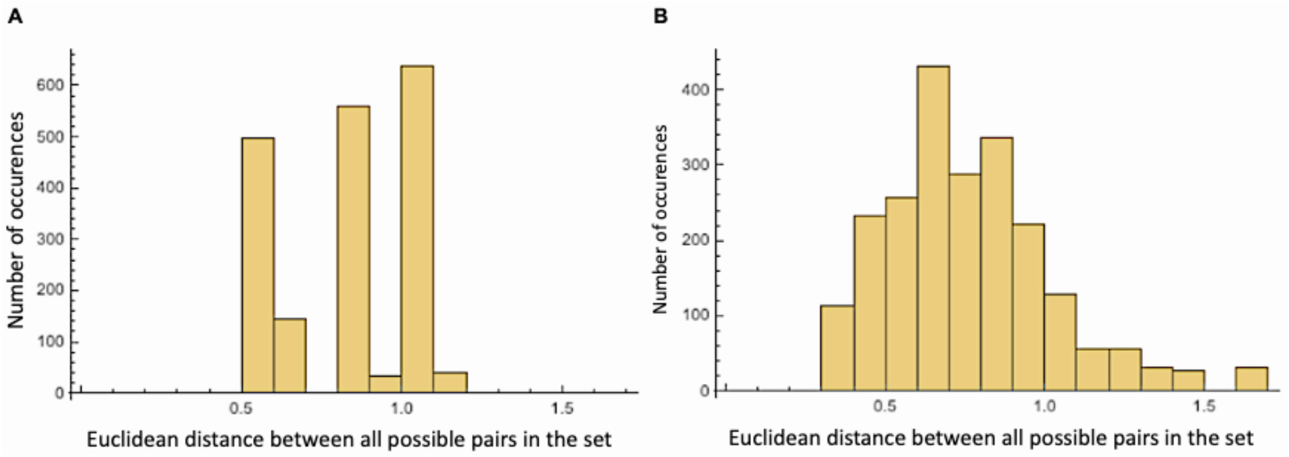

Figure S2. Distances within the two sets of *optimal* and *non-optimal* features, related to STAR Methods. (A) Distances within the set of *non-optimal* features. (B) Distances within the set of *optimal* features. The histograms of inter-feature distances taken within the two sets of features indicate the diameter of the respective feature sets in the 9-dimensional space of frequency spectra. The spread of frequency spectra within the set of *non-optimal* features [0, 1.2] is not much lower than the diameter of the entire feature space. For the optimal set of features, it is even the same as the whole space; all one can see is a slight tendency for lower values. These results show that the two sets of features do not occupy specific corners of the frequency spectrum but are rather spread over the entirety of the theoretically available space.

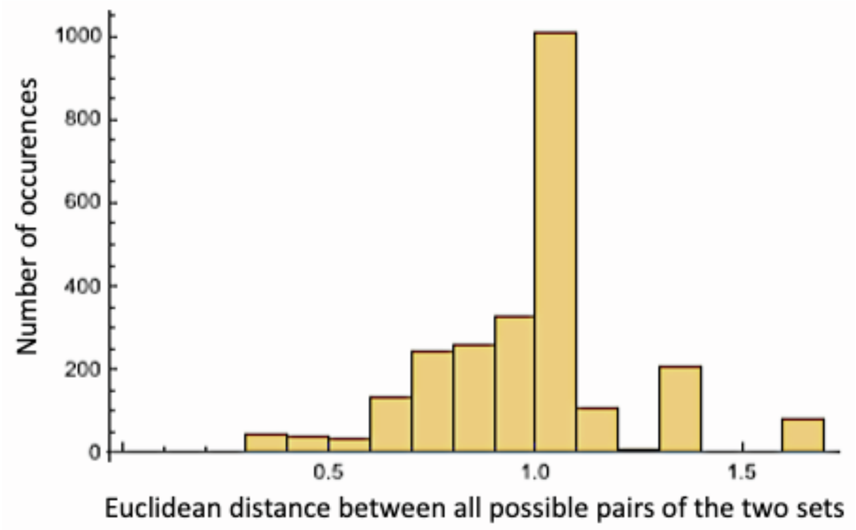

Figure S3. Distances between *optimal* and *non-optimal* feature sets, related to STAR Methods. The distribution of distances between all possible pairs of features, formed by picking one in each of the two sets (*optimal* vs. *non-optimal*), covers again the [0.3, 1.6] range. Comparing the means of individual components of the nine-dimensional spectra of the two sets, the resulting z-score was equal to or less than 1. In sum, the two sets have spectra that do not differ by much more than the typical distances within each individual set, and they both extend over essentially the whole frequency space theoretically allowed for their size. Also, by looking at the closest *non-optimal* feature to each of our *optimal* features, it turns out that, for 48 out of our 50 *optimal* features there is at least one *non-optimal* feature at a distance of less than 0.5 - that is less than the minimum distance between any pairs that can be formed between *non-optimal* features themselves. In light of these results, the different curvature effects induced by *optimal* vs. *non-optimal* features cannot be explained by their spatial frequency content.
